# Assessing the effects of malaria interventions on the geographical distribution of parasitaemia risk in Burkina Faso

**DOI:** 10.1186/s12936-016-1282-x

**Published:** 2016-04-21

**Authors:** Eric Diboulo, Ali Sié, Penelope Vounatsou

**Affiliations:** Swiss Tropical and Public Health Institute, Socinstrasse 57, P.O. Box, CH-4002 Basel, Switzerland; University of Basel, Basel, Switzerland; Centre de Recherche en Santé de Nouna, Nouna, Burkina Faso

**Keywords:** Malaria intervention effects, Geographical distribution, Parasitaemia risk, Burkina Faso, Bayesian geostatistical models

## Abstract

**Background:**

Burkina Faso conducted its first nationally representative household malaria survey in 2010/2011. The survey collected among others, information on malaria interventions, treatment choices and malaria parasite prevalence in children aged 6–59 months.

**Methods:**

In this study, Bayesian geostatistical models were employed to assess the effects of health interventions related to insecticide-treated bed nets (ITN), indoor residual spray (IRS), artemisinin-based combination therapy (ACT) coverage associated with childhood malaria parasite risk at national and sub-national level, after taking into account geographical disparities of climatic/environmental and socio-economic factors. Several ITN coverage measures were calculated and Bayesian variable selection was used to identify the most important ones. Parasitaemia risk depicting spatial patterns of infections were estimated.

**Results:**

The results show that the predicted population-adjusted parasitaemia risk ranges from 4.04 % in Kadiogo province to 82 % in Kompienga province. The effect of ITN coverage was not important at national level; however ITNs have an important protective effect in Ouagadougou as well as in three districts in the western part of the country with high parasitaemia prevalence and low to moderate coverage. There is a large variation in ACT coverage between the districts. Although at national level the ACT effects on parasitaemia risk was not important, at sub-national level 18 districts around Ouagadougou deliver effective treatment.

**Conclusion:**

The produced maps show great variations in parasitaemia risk across the country and identify the districts where interventions are being effective. These outputs are valuable tools that can help improve malaria control in Burkina Faso.

**Electronic supplementary material:**

The online version of this article (doi:10.1186/s12936-016-1282-x) contains supplementary material, which is available to authorized users.

## Background

Malaria is holoendemic in Burkina Faso with most transmission occurring during or shortly after the rainy season between July to December. Ninety-nine percent of infection is attributed to *Plasmodium falciparum.* The overall prevalence of infection in children aged 6–59 months is estimated at 66 % [Burkina Faso Demographic and Health Survey-Multiple Indicator Cluster (BFDHS-MICS 2010)] [1]. The under-five severe malaria attributable death has been dropped from 8.1 % in 2000 to 3.3 % in 2010 [2]. The government has made tremendous efforts to achieve the objectives of the 2006–2010 National Malaria Strategic Plan and implemented special programmes, such as home-based malaria management in 2008, universal coverage of insecticide-treated bed nets (ITN) in 2010, intermittent preventive therapy (IPT) for high-risk groups in 2005, piloting of the indoor residual spray (IRS) in certain health districts since 2010, larval control and sanitation programmes, introduction of effective tools for malaria control mainly the rapid diagnostic test (RDT) at all health facilities in 2010 and actual availability of artemisinin-based combination therapy (ACT) in health facilities in 2007 [2].

Burkina Faso carried out the first malaria indicator survey (MIS) in 2010, a nationally representative household survey in the country compiling malaria-related indicators. MIS surveys generate a number of indicators of malaria intervention coverage that can be used to assess progress towards the goals of the global malaria action plan (GMAP) [3]. These indicators measure ownership, use and access of ITNs, implementation of IRS, access to ACT and to intermittent preventive treatment for pregnant women (IPTp). MIS data have been used to assess effects of interventions [4–10]. Some studies reported protective effects [4, 7] for specific interventions and others did not find any effect [9, 10]. In the analysis of the Senegal MIS data of 2008, using geostatistical variable selection, it appeared that among the various indicators of ITN ownership, only few were able to estimate a protective effect of ITN intervention on parasitaemia risk [4].

Intervention effects are likely to vary in space because there is often geographical variation in the intervention coverage and their effects are related to malaria endemicity [11–13]. Recently, the effects of vector-control interventions on changes of malaria parasite risk were estimated at different spatial resolutions in six sub-Saharan African countries using Bayesian geostatistical models with spatially varying covariates. Results suggested that some interventions may not show any effect when looked upon at national level while they can have a protective effect at sub-national level [7].

Samadoulougou et al. [6] used the Burkina Faso MIS 2010 data to estimate the spatial distribution of malaria risk among children under five of age in Burkina. The authors included only ITN use as an intervention-related predictor and estimated an overall effect at country level, which was not statistically significant.

In this study, MIS data were analysed using Bayesian geostatistical models to assess the effects of different malaria interventions at national as well as sub-national level (50 health districts) in the country. Bayesian variable selection within geostatistical models allowed to screen different coverage measures for each intervention and spatially structured regression coefficients measured the effects of interventions at district level. Predictive maps of the disease burden adjusted for climatic effects were also produced.

## Methods

### Country profile

Burkina Faso lies mostly between latitudes 9° and 15°N and longitudes 6°W and 3°E. It is made up of two major types of countryside. The larger part of the country is covered by a “peneplain”, which forms gently undulating landscapes with, in some areas, a few isolated hills. The southwest of the country forms a sandstone massif bordered with sheer cliffs up to 150 m high. Burkina is a relatively flat country with an average altitude of around 400 m. Four main rivers drain the country: the *Mouhoun*, the *Nakambé*, the *Nazinonand* the *Komoé*. The Mouhoun is one of the country’s only two rivers which flow year-round, the other being *Komoé*, which flows to the southwest. Burkina Faso has a primarily tropical climate with two very distinct seasons. In the rainy season, the country receives between 600 and 900 mm of annual rainfall and malaria is known for a seasonal recrudescence during this period at which it accounts for the main cause of fever and mortality in the country. The rainy season lasts approximately 4 months, May/June to September, and is shorter in the north of the country. In the dry season, the *harmattan*, a hot dry wind blows from the Sahara carrying dust and dirt that contribute to high morbidity from lower respiratory infections.

### Survey data

The MIS was conducted by the National Institute for Statistics and Demography (INSD) with the technical assistance of ICF Macro from April 2010 to January 2011 using standardized malaria indicator questionnaire. The collected data include information on malaria indicators, education, demographics, and socio-economic characteristics.

**Fig. 1:**
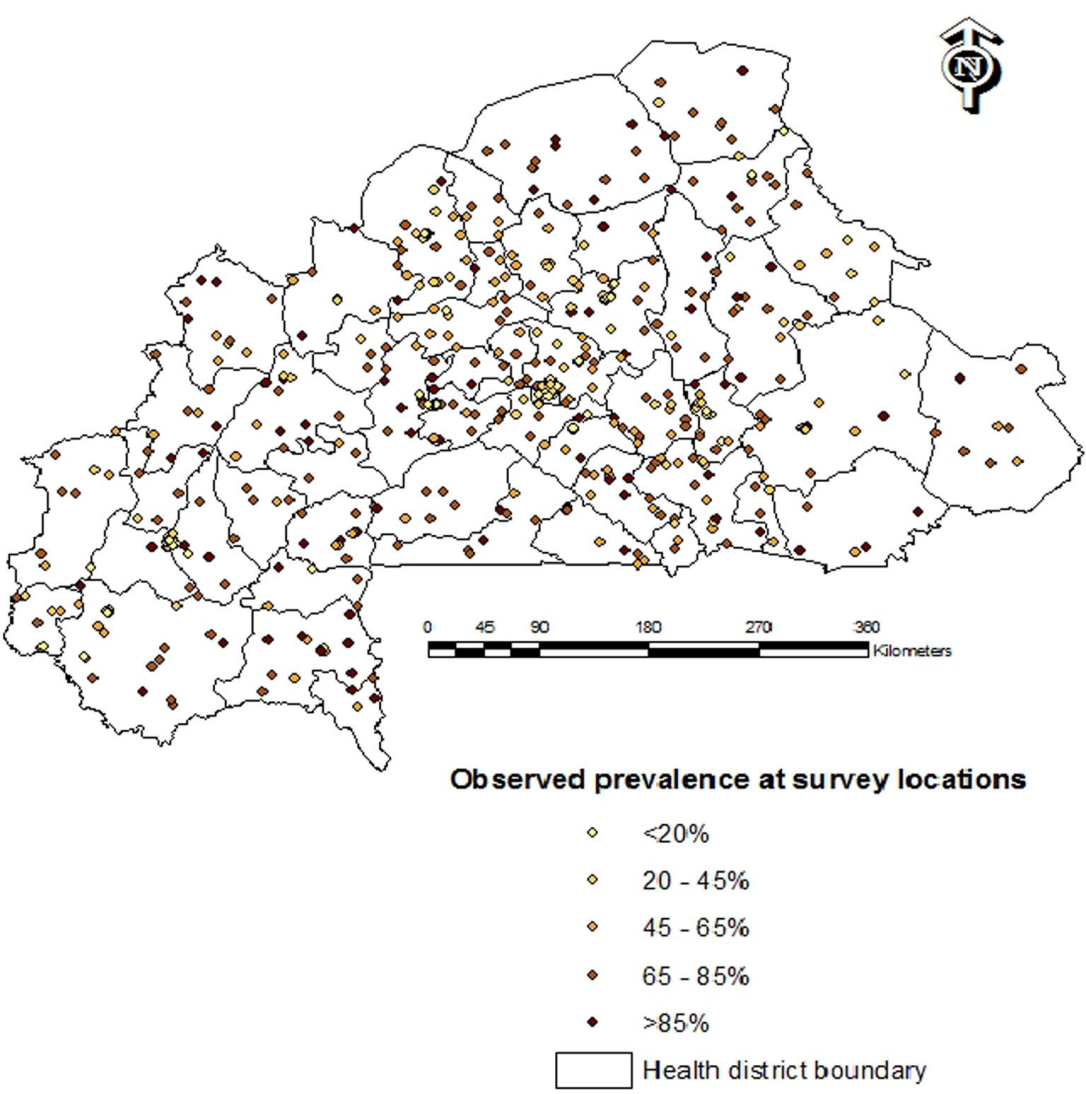
Observed prevalence at survey locations, Burkina Faso MIS 2010

A random sample of 574 (176 and 398 respectively in urban and rural settings) clusters and 15,000 households were selected through a stratified two-stage sampling procedure. The clusters were the census units established by INSD in the census carried out in 2006 (Récensement Général de la Population et de l’Habitat, RGPH-2006). At the first stage, 574 clusters were drawn with probability proportional to the number of households in each cluster. The sampling procedure was stratified by area type (urban/rural) of the cluster and by the administrative regions (13 regions). At the second sampling stage, a count of households in each of these 574 clusters provided a list of households from which was derived the final households sample with an equal probability systematic sampling. As part of the final sampling, one in every two households was randomly selected and every child between 6 and 59 months of age was tested for parasitaemia. Two malaria diagnostic tests were performed, namely RDT and blood smear test (microscopy). Analyses in this study are based on the results of microscopic examination since it is considered as the gold standard [14]. Geographic information was collected at the centroid of the clusters. Figure 1 shows the observed prevalence reported in 540 survey locations.

The MIS data were used to construct intervention coverage measures related to ITN such as the number of ITNs per child under 5 years (ITNpU5), the proportion of children under five who slept under an ITN the night preceding the survey (ITNsU5) and the total number of ITNs per household member (ITNpPR). An indoor residual spray (IRS) coverage indicator was defined as the proportion of households sprayed with an insecticide within the last 12 months. A ‘case management’ coverage measure was generated as a proxy of health system performance. It was defined by the proportion of children under five in the cluster who had received timely a first line ACT out of those reported to have fever 2 weeks prior the survey visit (ratio of ACT per reported recent fever).

Socioeconomic status (SES) was captured by mother’s education and wealth index. The latter was calculated as a weighted sum of household assets; the weights were obtained through principal component analysis [15]. Socioeconomic quintiles ranging from the poorest to the wealthiest were included as a categorical variable in the analysis.

### Climatic data

Malaria transmission is environmentally driven; therefore remotely sensed (RS) climatic and environmental proxies were used as predictors in the models to take into account their potential effects on parasitaemia. In particular, the following climatic and environmental factors were included in the models: land surface temperature day (LSTD) and night (LSTN), rainfall estimates (RFE), normalized difference vegetation index (NDVI), altitude, urban–rural extent, proximity to the rice field (within 5 km radius from the survey locations) and the shortest Euclidean distance to nearest water body calculated in ArcGIS 10 (ESRI; Redlands, CA, USA). Table 1 indicates the sources of these data as well as their spatial and temporal resolution.

**Table 1:** Environmental and climatic data

Source	Data	Period	Spatial resolution	Temporal resolution
Moderate resolution imaging spectroradiometer (MODIS) terra	Day and night land surface temperature (LST)	2010–2011	1 × 1 km^2^	8 days
Moderate resolution imaging spectroradiometer (MODIS) terra	Normalized difference vegetation index (NDVI)	2010–2011	0.25 × 0.25 km^2^	8 days
Afripop	Population data	2010	1 × 1 km^2^	NA
Africa data disseminating services	Rainfall	2010–2011	8 × 8 km^2^	10 days
Digital elevation model (altitude)	Shuttle radar topographic mission (SRTM)	2000	1 × 1 km^2^	NA
National database of land use	Rice cultivation field	2002	0.25 × 0.25 km^2^	NA
Health mapper	Water bodies	–	1 × 1 km^2^	NA
Global rural and urban mapping project	Urban rural extent	2010	1 × 1 km^2^	NA

### Bayesian geostatistical modelling

Three Bayesian geostatistical logistic regression models were fitted, firstly to estimate the geographical distribution of malaria risk based on climatic predictors (model 1); secondly to assess the effects of malaria related interventions (i.e. ITN, IRS and ACT coverage) at national level after adjusting for climatic and socio-economic (i.e. wealth index, mothers education) confounders (model 2) and thirdly to assess the effects of the above mentioned interventions at the level of the health district (model 3). The climatic model (model 1) was fitted on the number of malaria-infected children at cluster level. The models with intervention effects (models 2 and 3) were applied on the binary outcome indicating the infection status of a child and considering the age of a child (in years) as a covariate. All models included cluster-specific random effects, arising from a Gaussian stationary process with covariance matrix capturing correlation between any pair of cluster locations as a function of their distance. Bayesian geostatistical variable selection was applied in model 1 to identify the most important set of climatic predictors (including their best functional form) [16]. In particular, for each climatic predictor an indicator was introduced to estimate the probabilities of excluding or including the predictor into the model in linear or categorical form. The categories of the predictors were defined using their quartiles. The final model included variables and functional forms with inclusion probability of more than 50 %. Model 2 contained the most important climatic/environmental predictors selected in model 1 as well as age, SES, ITN, IRS and ACT coverage measures. Geostatistical variable selection was applied to select the best ITN coverage measure (i.e. ITNpU5, ITNsU5 and ITNpPR) by introducing binary indicators specifying the exclusion or inclusion of each measure from the model.

The effects of malaria interventions (i.e. ITN, IRS and ACT coverage) at sub-national level were estimated from the geostatistical model 3 which includes the intervention coverage measures as spatially varying covariates, following model formulations used by Giardina et al. [7]. A conditional autoregressive (CAR) prior distribution [17] was considered to introduce a neighbour-based spatial structure on the regression coefficients related to each intervention effect in the study [7, 18]. Neighbours were defined as the adjacent areas (health districts) of each health district and used to create a matrix of spatial weights taking the value of one for a direct neighbour and zero otherwise. Model 3 utilizes the ITN coverage measure, which was selected in model 2.

Bayesian kriging [19] was employed to predict the malaria parasite risk over a regular grid of 226,627 pixels at 1 km^2^ resolution covering the entire study area. Population adjusted risk estimates were obtained at district level by combining pixel-level risk estimates with population data of children under 5 years. The analysis was carried out in STATA 13 (Stata corporation, College Station, Texas, USA) and OpenBUGS version 3.2.3 rev 1012 (Imperial College and Medical Research Council, London, UK). Parameter estimates were summarized by their posterior median and the corresponding 95 % Bayesian credible interval (BCI). Modelling details are given in Additional file 1.

The predictive model was validated on a test subset of the data. A randomly selected sample of 432 locations (80 % of the data location) was used as a training set for the model fit. The predictive performance of the model was assessed by calculating the proportion of observed prevalence data at the remaining 20 % of the test locations, correctly estimated within Highest Posterior Density Intervals (HPDI) of probability coverage ranging from 50 to 95 %.

## Results

A total of 5741 children aged between 6 and 59 months from 574 clusters were tested for parasitaemia using blood smear test results. The overall observed malaria parasite prevalence was 65 %. Thirty-four (5.92 %) clusters had no data thus reducing the actual number of clusters to 540 (Fig. 1).

The ratio of ITN to children under 5 years old ranges from 0.33 in district of Orodara to 1.52 in Nanoro district with 11 out of the 50 districts in the country having a ratio above 1. 80 % of the 50 health districts have IRS coverage less than 1 %. The maximum IRS coverage of 50 % was found in the districts of Diebougou and Toma. The percentage of households falling in the lowest social-economic quintile ranges from 1.6 % (in Tenkodogo) to 63.33 % (in Gorom-gorom). Table 2 gives a summary of the raw coverage measures per health district. The highest proportion of fever cases receiving ACT is 22 % and it is observed in the districts of Tenkodogo in the East central and Koudougou in the Central part of the country. Figure 4 (right) depict the ITNpU5, ITNsU5 and ACT coverage at health district level, respectively.

**Table 2:** Summary of raw malaria coverage measures per health district

Health district	Number of clusters	ITNpU5 (%)	ACT (%) (# of fever cases)	IRS (%) (# of clusters)	ITNsU5 (%)	HH in lowest quintile (%)
Banfora	26	109	3 (50)	4 (1)	76	4.37
Barsalogho	6	50	2 (9)	0 (0)	50	23.94
Batie	4	73	1 (7)	0 (0)	38	59.26
Bogande	14	63	1 (26)	0 (0)	54	44.91
Boromo	4	67	0 (16)	0 (0)	56	8.93
Boulsa	9	50	3 (14)	0 (0)	47	13.04
Bousse	6	95	3 (5)	0 (0)	80	24.00
Dande	7	64	1(17)	0 (0)	58	10.00
Dano	12	61	4 (38)	0 (0)	43	47.62
Dedougou	11	73	3 (23)	0 (0)	56	15.53
Diapaga	9	121	0 (6)	0 (0)	63	59.46
Diebougou	4	133	8 (14)	50 (2)	33	14.63
Djibo	15	49	6 (33)	0 (0)	53	39.35
Dori	11	92	2 (11)	0 (0)	60	44.00
Fada N’gourma	17	97	3 (24)	0 (0)	73	28.48
Gaoua	14	109	4 (52)	0 (0)	71	47.76
Gorom-gorom	9	46	11 (14)	0 (0)	38	63.33
Hounde	5	78	0 (21)	0 (0)	50	9.33
Kaya	18	37	12 (4)	0 (0)	39	14.18
Kombissiri	6	46	10 (33)	0 (0)	20	12.50
Kongoussi	10	57	2 (6)	0 (0)	42	23.08
Koudougou	14	68	21 (38)	7 (1)	23	7.24
Koupela	15	84	10 (16)	0 (0)	58	26.04
Leo	7	66	9 (16)	0 (0)	62	14.63
Manga	13	51	2 (34)	0 (0)	21	18.52
Nanoro	4	152	9 (10)	0 (0)	100	14.29
Nouna	10	63	2 (22)	0 (0)	57	13.04
Orodara	9	33	3 (31)	0 (0)	24	20.00
Ouagadougou	36	98	13 (45)	6 (2)	42	1.16
Ouahigouya	24	136	4 (51)	0 (0)	90	15.15
Ouargaye	4	60	1 (4)	0 (0)	17	22.86
Pama	6	86	1 (8)	17 (1)	50	32.95
Po	12	54	4 (18)	0 (0)	42	31.67
Reo	11	75	6 (17)	0 (0)	55	37.23
Sapone	10	60	7 (14)	0 (0)	22	3.28
Sebba	9	87	1 (6)	11 (1)	50	51.11
Secteur 15	13	55	7 (29)	0 (0)	67	7.96
Secteur 22	12	62	8(39)	0 (0)	60	6.84
Seguenega	6	121	1 (25)	0 (0)	53	5.00
Sindou	7	68	2 (31)	0 (0)	63	7.23
Solenzo	8	85	13 (15)	0 (0)	64	20.48
Tenkodogo	18	51	22 (60)	0 (0)	58	1.06
Titao	5	116	1 (20)	0 (0)	58	25.37
To	8	66	5(22)	0 (0)	30	23.46
Toma	4	55	1 (19)	0.5(2)	67	3.64
Tougan	9	56	0 (26)	22 (2)	24	2.02
Yako	10	142	3 (31)	0 (0)	65	19.54
Zabre	4	87	7 (10)	0 (0)	88	52.08
Ziniare	19	104	5 (48)	0 (0)	49	15.00
Zorgho	15	140	11 (74)	7 (1)	91	11.68

ITNpU5 over 100 % indicate more than one net per person

The results of variable selection are presented in Table 3. In particular, Bayesian geostatistical variable selection applied in the climatic model 1 indicated that the most important factors related to parasitaemia risk are LSTN (Night Land Surface Temperature) in linear form and the proximity to rice cultivation (categorical) with 74.10 and 74.50 % posterior inclusion probabilities, respectively. The place of residence (rural/urban) stood out as one of most important predictors with a posterior inclusion probability of 87.20 %. Geostatistical variable selection of the ITN coverage measures (model 2) showed that the proportion of children that slept under a net (ITNsU5) had the highest probability (equal to 32.02 %) to be included in the model among the ones that were assessed. The inclusion probability of less than 50 % indicates that ITNsU5 is less likely to have an important effect at national scale. However, ITNsU5 was included in model 3 as a spatially varying covariate to assess important effects at sub-national levels and identify health districts that the ITN interventions are associated with the parasitaemia risk.

**Table 3:** Results of variable selection for the climatic predictors and ITN coverage measures based on Bayesian geostatistical logistic regression models

Posterior inclusion probability (%)		
Variable	Model 1	Model 2
Altitude	28	
Distance to water body	2	
NDVI	3.30	
LSTD	6.20	
LSTN	74.10	
Rainfall	1.60	
Distance to rice growing area	0.30	
Altitude^a^	5.70	
Distance to water body^a^	4	
Area type (urban/rural)^a^	87.20	
NDVI^a^	0.20	
LSTD^a^	4.50	
LSTN^a^	3.50	
Rainfall^a^	1	
Distance to rice growing area^a^	74.50	
ITN coverage		
ITN per person (ITNpPR)	–	0.20
U5 sleep under net (ITNsU5)	–	32.02
ITN per under 5 years (ITNpU5)	–	2.46

Posterior inclusion probabilities larger than 50 % indicate an important predictor

^a^Categorical form

**Fig. 2:**
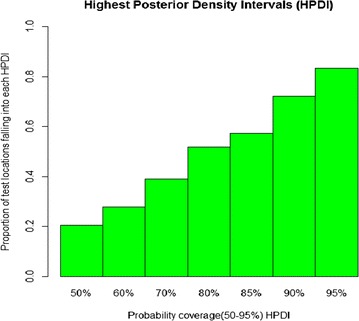
Proportion of test locations falling in the Highest Posterior Density intervals (HPDIs)

The predictive performance of the model (model2) is shown in Fig. 2. Eighty-two (82 %) of test locations were falling into all credible intervals with probability areas greater than 50 %.

Parameter estimates of the three models are given in Table 4. In particular the climatic model 1 showed a negative correlation of the parasitaemia risk with LSTN and distance to rice fields. Moreover, living in rural areas increases the odds of being infected by about 3.91 times (95 % BCI: 2.88, 5.22). The minimum distance at which the spatial correlation is less than 5 % is equal to 10.61 km (95 % BCI: 2.39–20.82). Model 2 assesses the effects of malaria interventions on parasite risk after adjusting for climatic and socio-economic confounders. Results show that none of the health intervention measures is an important predictor of parasitaemia risk at national level. However, the model indicates that the odds of malaria infection decreases with better socioeconomic conditions reaching a 68 % reduction within the least poor group, OR = 0.32 (95 % BCI: 0.24–0.43). An increasing gradient of malaria risk was also observed with age with the oldest age group (4–5 years old) having odds of 2.08 times higher than infants. Furthermore, the model reveals a decreasing trend of parasitaemia odds with increasing mother’s education level although this decrease is not statistically important. The additional SES and malaria interventions related predictors in model 2 were able to explain some of the spatial correlation in the model. This is reflected in the estimate of the range parameter, which reduced to 3 km compared to 10.6 km in model 1.

**Table 4:** Posterior median and 95 % Bayesian credible intervals (BCI) of the geostatistical model based on environmental/climatic, malaria intervention coverage and SES predictors

	Geostatistical model 1	Geostatistical model 2	Geostatistical model 3
Variables	OR (95 % BCI)	OR (95 % BCI)	OR (95 % BCI)
LSTN	0.78 (0.69, 0.88)	0.81 (0.72, 0.90)	0.82 (0.72, 0.93)
Rice field proximity	0.49 (0.27, 0.81)	0.56 (0.34, 0.98)	0.73 (0.43, 0.99)
No	1.00	1.00	1.00
Yes	0.49 (0.27, 0.81)	0.56 (0.34, 0.98)	0.73 (0.43, 0.99)
Area type			
Urban	1	1	1
Rural	3.91 (2.88, 5.22)	2.50 (1.89, 3.35)	2.36 (1.79, 3.13)
SES			
Most poor		1	1
Very poor		0.76 (0.61, 0.95)	0.77 (0.61, 0.96)
Poor		0.90 (0.71, 1.14)	0.92 (0.73, 1.16)
Less poor		0.70 (0.55, 0.89)	0.71 (0.56, 0.90)
Least poor		0.32 (0.24, 0.43)	0.33 (0.25, 0.45)
Age (years)			
0–1		1	1
1–2		1.56 (1.23, 1.56)	1.21 (0.95, 1.55)
2–3		1.72 (1.35, 2.21)	1.71(1.33, 2.19)
3–4		1.88 (1.48, 2.41)	1.87 (1.47, 2.39)
4–5		2.08 (1.62, 2.67)	2.07 (1.61, 2.65)
Mother’s education			
No education		1	1
Primary		1.08 (0.89, 1.31)	0.85 (0.71, 1.03)
Secondary		1.06 (0.74, 1.54)	0.88 (0.61, 1.27)
Higher		1.44 (0.29, 6.49)	0.42 (0.05, 2.13)
Case management (ACT)	–	1.45 (0.49, 4.21)	0.13 (−1.49, 1.67)
U5 sleep under net (ITNsU5)	–	1.66 (0.89, 3.08)	0.25 (−0.37, 0.90)
House Spray (IRS)	–	1.14 (0.17, 7.23)	0.11 (−1.75, 1.70)
Variances			
Gaussian process	0.87 (0.66, 1.15)	0.6 (0.44, 0.80)	0.55(0.39, 073)
Spatially varying ITNsU5			0.57 (0.38, 0.88)^b^
Spatially varying IRS			0.76 (0.42, 1.75)^b^
Spatially varying ACT			0.75 (0.42, 1.84)^b^
Range (km)^a^	10.61 (2.39, 20.82)	3.00 (0.40, 10.00)	2.70 (0.44, 9.75)

Model 1 includes only climatic factors

Model 2 includes climatic factors, age, SES, intervention measures

Model 3 has spatially varying covariates for IRS, ACT and ITNsU5

^a^Minimum distance in kilometer at which the spatial correlation is lower than 5 %

^b^Posterior median

**Fig. 3:**
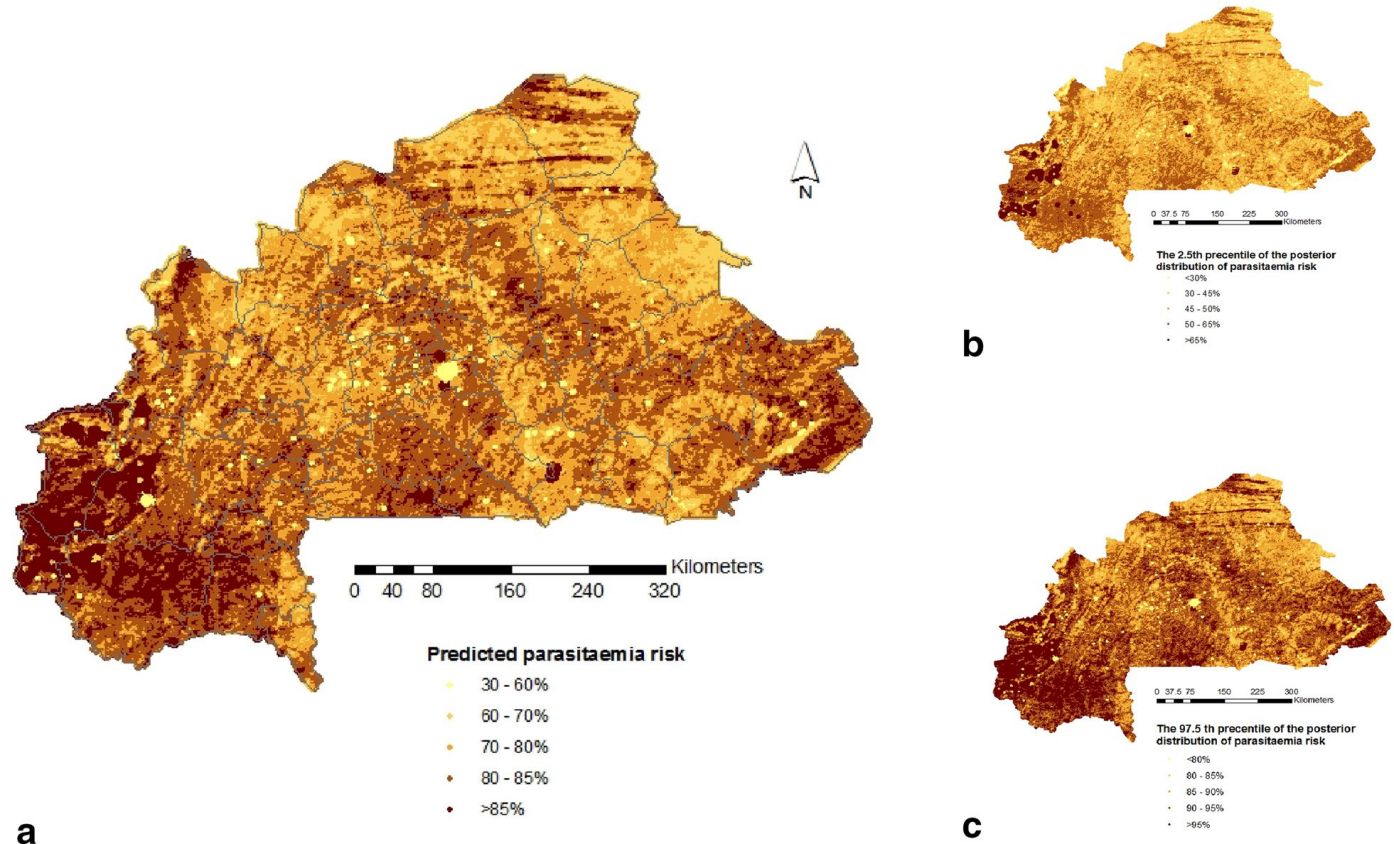
Predicted parasitaemia risk map in children under 5 years old based on the **a** median **b** 2.5th percentiles and **c** 97.5th percentiles of the posterior predictive distribution estimated from model 2 at 1 km^2^ resolution. Province boundaries are overlaid

Figure 3a–c depict the predicted parasitaemia risk maps (median (a), 2.5th (b) and 97.5th (c) percentiles of the posterior predictive distribution estimated from model 2) in children less than 5 years of age at 1 km^2^ spatial resolution in Burkina Faso. Estimates show that malaria parasitaemia risk ranges from 36 to 71 % across the country while the median predicted prevalence is 59 %. The Southwest, Comoe, Cascade, East, Central-west, Boucle du Mouhoun and the Sahel regions bear the highest prevalence. The central, the North, the east central and regions appear to be the less burdened regions. The total number of infected children under 5 years old in the country was estimated to be 1097,296. Table 5 presents the population-adjusted and estimated number of infected under five children under 5 years of age per province and region.

**Table 5:** Population-adjusted and estimated number of infected children under 5 years old per province and region

Region	Province	Observed prevalence (%)	Population of children	Population adjusted estimated prevalence	Estimated number of infected children
Boucle du Mouhoun	Bales	79	37,601	17.72	17,646
	Banwa	78	47,308	16.92	21,975
	Kossi	76	48,528	28.96	18,321
	Mouhoun	74	52,335	20.85	21,159
	Nayala	81	28,425	19.12	13,828
	Sourou	63	38,168	21.79	18,577
Cascades	Comoe	59	71,772	33.13	32,656
	Leraba	55	20,765	25.44	8000
Centre	Kadiogo	34	243,402	4.04	139,826
Centre-Est	Boulgou	62	98,226	10.91	41,548
	Koulpelogo	75	47,315	18.04	18,495
	Kouritenga	52	59,702	7.44	24,678
Centre-Nord	Bam	60	50,553	11.46	24,229
	Namantenga	78	60,409	16.08	27,146
	Sanmatenga	62	109,630	13.10	46,970
Centre-Ouest	Boulkiemde	64	87,305	8.20	34,159
	Sanguie	88	51,241	14.39	24,188
	Sissili	75	37,038	28.00	16,542
	Ziro	77	31,847	27.71	12,820
Centre-Sud	Bazega	67	39,878	15.11	17,614
	Nahouri	71	27,251	20.69	11,722
	Zounweogo	75	42,191	13.65	18,243
Est	Gnagna	74	78,380	18.22	31,657
	Gourma	66	59,266	33.75	22,215
	Komandjari	41	15,841	47.24	7041
	Kompienga	73	15,690	81.72	5842
	Tapoa	67	67,016	38.67	25,250
Haut-Bassins	Houet	52	148,488	10.68	76,584
	Kenedougou	65	44,522	25.62	20,182
	Tuy	75	35,504	21.55	18,267
Nord	Loroum	72	25,989	20.06	12,062
	Passore	56	57,802	9.67	25,994
	Yatenga	66	99,943	9.80	47,657
	Zandoma	60	30,413	8.98	13,394
Plateau central	Ganzourgou	66	56,713	10.46	27,228
	Kourweogo	52	24,218	9.90	11,251
	Oubritenga	65	42,149	10.15	18,400
Sahel	Oudalan	72	35,453	46.15	14,507
	Seno	63	47,034	19.54	23,291
	Soum	85	62,279	31.43	26,560
	Yagha	51	28,730	34.73	12,464
Sud-Ouest	Bougouriba	59	17,941	24.32	7492
	Ioba	83	33,185	15.01	14,889
	Noumbiel	87	12,456	36.51	5086
	Poni	76	45,305	26.78	19,638

**Fig. 4:**
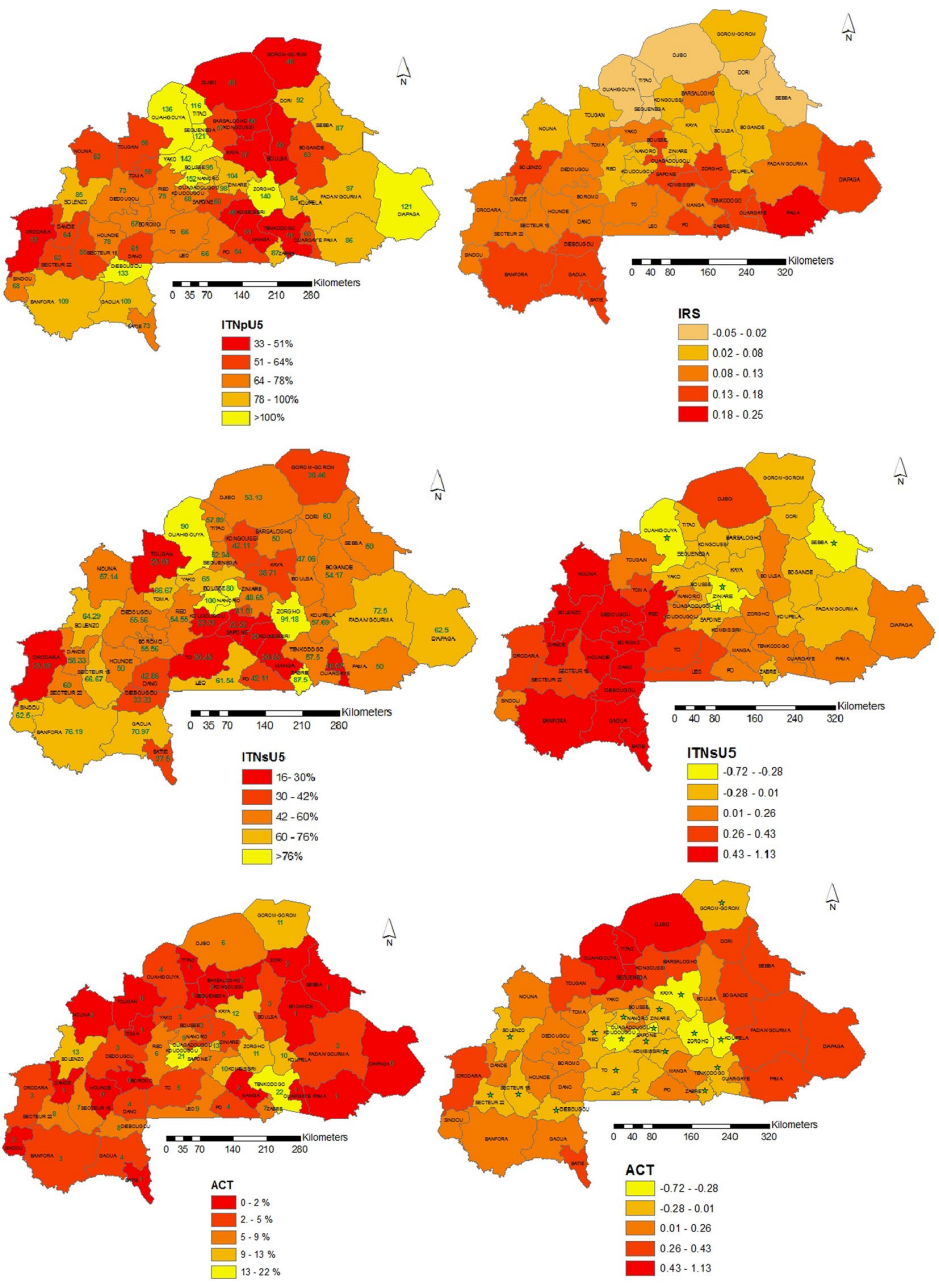
Coverage of ITNpU5, ITNsU5 and ACT (*right*) and IRS, ITNsU5 and ACT intervention effect maps (*left*). Important effects are indicated with (*) and correspond to 95 % Bayesian credible intervals that do not include 0

Spatially structured coefficients of intervention coverage measures obtained by model 3 allowed estimation of the effect of ITN, IRS and ACT at health district level. Figure 4 presents the different coverage (right hand side maps) and intervention effects (left hand side maps) for each health district estimated from model 3 with the spatially varying covariates. ACT coverage appears to be an important health system component associated with decreased malaria parasitaemia risk in a number of the health districts. However the strongest effects are observed in the districts of Ouagadougou, Koudougou, Kaya, Zorgho, Koupela and Tenkodogo. ITN usage shows a protective effect in only four health districts namely Ouagadougou, Ziniare, Ouahigouya and Sebba. IRS which is in a pilot phase in the district of Diebougou (South-west) did not show any effect.

## Discussion

This is the first study in Burkina Faso to estimate the spatial effects of malaria interventions on the geographical distribution of parasite risk. Different ITN coverage measures related to bed net use and ownership were calculated and the effects of ITN, IRS and case management coverage on the spatial pattern of malaria risk were studied after taking into account disease variation due to climatic and socio-economic factors. Georeferenced estimates of the parasite risk were obtained as well as the number of infected children. This study analysed the Burkina Faso MIS survey data of 2010 and employed Bayesian geostatistical models with spatially varying covariates using geostatistical variable selection to identify the most relevant bed net coverage indicators. The geostatistical variable approach identified a list of the most important climatic and environmental predictors. Weiss et al. [20] have proposed a list potential predictors that could be used to improve malaria modelling.

The most important ITN coverage measure was the proportion of children under 5 years old sleeping under bed net, with however a low posterior probability to be included in the model equal to 32.02 % followed by far by the number of ITN per under five (2.46 %) and the number of ITN per household member (0.20 %). This finding support previous results showing that contingent upon the setting and the prevailing conditions, ITN ownership and ITN use may show different ability in capturing ITN intervention effects on parasitaemia [4].

Overall, the effect of interventions in Burkina Faso was not significantly associated with change in malaria parasitaemia risk at country level. These results are in line with the findings of Giardina et al. [7] in a multisite study. The lack of statistically important ITN intervention effects may be explained by the fact that at country-level, a sizeable percentage (37.97 %) of children under the age of 5 years still do not sleep under ITN. However when analysed at sub-national level (health district level) some interventions show protective effects in certain districts. For instance ITN use, which appeared to have not an important effect at national level proved to be an effective intervention in four health districts namely Ouagadougou, Ziniare in the central region of the country, Ouahigouya in the North and Seeba in the Sahel region. Among factors that might affect ITN effectiveness feature the inadequate coverage and the uneven distribution of ITN and the usage among households and health districts [21].

Indeed, malaria is holoendemic in Burkina Faso and the transmission occurs throughout the year. In such settings, even significant reductions in the total exposure would not necessarily warrant substantial reductions in parasitaemia [22–25]. Furthermore, there is still low ITN utilization compliance (62.03 %) among children under the age of 5 years. This study found a protective effect of ITN usage in specific districts. This result supports findings reporting that the ITN intervention is expected to be more effective in low transmission settings with the highest ITN usage [26]. Districts where ITN use was found to be protective are located in low to mild transmission settings where coverage of ITN use ranges from 42 to 90 % [27].

ACT showed a protective effect in 19 health districts. However it is worth noting that except Gorom-gorom in the Sahel region, Solenzo in the North West (Boucle du Mouhoun), secteur 15 and 22 in the West (Hauts-bassins) and Diebougou in the Southwest, the remaining districts where ACT showed a protective effect are located in the central region. A close inspection of the coverage levels shows that ACT tends to be effective in districts where a minimum level of coverage is achieved (above 5 %). This finding supports the hypothesis that the effectiveness of a given intervention is related to both its coverage as well as the transmission levels. Indeed, the ACT effect is presumably related to the very low coverage [28]. High levels of transmission are also believed to limit the effect of ACT. Findings from a study conducted in Tanzania suggested that the percentage reductions in prevalence of infection and incidence of clinical episodes achieved by ACT were much higher in areas with low initial transmission [28]. ITN interventions, however, aim at reducing the malaria transmission intensity by reducing the chances that an individual will be bitten by an infective *Anopheles* mosquito [29]. However low compliance in ITN use may seriously reduce the potential impact of ITNs [27, 30]. Therefore, conjugated efforts to increase both the ACT coverage (in order to reduce the prevalence) and ITN use (to further reduce the transmission) are required to warrant a synergetic effect towards a better and effective control of the disease [31]. Findings from a continental study that used data from 32 malaria-endemic African countries showed that ITN intervention was the most important and effective malaria intervention accounting for an estimated 68 % decline in malaria parasite rate in 2015 [32]. The geostatistical model was able to identify districts with important protective ITN effects, although at country level the effect was not important.

IRS was not associated with malaria parasitaemia risk most probably owing to an extremely low percentage of houses sprayed within the last 12 months (0.92 %) prior to the survey.

Malaria is known to be a climate-driven disease and among the most important climatic factors features temperature. The model-based parasitaemia risk map depicts a strong spatial with lower parasitaemia risk estimated in the cities (urban settings) relative to rural settings. The results show a negative association between increased night temperature and malaria transmission. Laboratory experiments observed the shortest *Anopheles gambiae s.s* larval survival (<7 days) at 10–12 and 38–40 °C and the highest larval mortality occurring between 30 and 32 °C, with death (rather than adult emergence) representing over 70 % of the terminal events in mosquitoes originally from Lagos (Nigeria) [33]. In Burkina Faso, the monthly mean temperature in the hottest and driest period (March–May) is constantly well above 31 °C. Land surface night temperature, therefore appears to be an important predictor of malaria transmission. Furthermore, the behavioural high temperature avoidance experiment showed that *An. gambiae,* the most efficient malaria vector species in Burkina Faso, was more sensitive to increased temperatures than its sibling species, *Anopheles arabiensis* [33]. In nature, this probably results in short distance flights to seek cooler spots, typically the shaded resting sites under vegetation outdoors or cool dark comers indoors. The highly endophilic nature of *An. gambiae* protects the mosquito from the highly variable and more extreme external climate. This may explain the negative association between increased LSTN and the transmission because during the hot night spells local populations rest outdoor thus reducing knowingly or unknowingly the contact human-vector. The authors also found that female temperature avoidance was most pronounced in hungry females (which avoid temperatures above 25 °C), less strong in blood-feds (above 30 °C) and least strong in newly emerged females (above 32 °C). High night temperatures were also found to affect *An. gambiae* (one of the most predominant and effective malaria vector in Burkina Faso) behaviour and vectorial capacity [34, 35]. A significant negative association between temperature and malaria infection was also found in a previous study in Burkina Faso [6]; However the authors did not consider day and night temperatures separately and the climatic data considered in this study do not span the study period (April 2010–January 2011). The map of nighttime land surface temperatures (LSTN) is also provided (see Additional file 2).

The present study estimated a negative association between malaria parasitaemia and proximity with rice growing areas. The rice growing areas used in this study were extracted from the National land use database with cartographic scale coverage of 1/200,000 which features only large and economically relatively important rice growing areas. Furthermore, as exposed to an increased risk of malaria infection, surrounding populations receive relatively high attention from the local government including regular sensitization campaigns (Information Education and Communication). Consequently, as an income generating activity, the local population is relatively well off. This makes it easier to access health care and other protective measures. This effect is known in Burkina Faso as the “paddy paradox” defined as the occurrence of large populations of vectors but low amounts of malaria transmission where irrigated rice is grown.

The negative association has been reported in other studies in Burkina Faso, Ghana, Gambia and Tanzania [36–39] The potential explanation might be that the irrigated rice fields are preferred habitats for *An. arabiensis,* which has a lesser vectorial capacity than other species [36].

Furthermore it is hypothesized that the “paddy paradox” is due to young pre-gravid mosquitoes dispersing more widely than gravid ones, not necessarily to low survival in the mosquito [37]. The map of the distance between the clusters and the nearest rice-paddy field in kilometre is also provided (see Additional file 3).

The predicted spatial distribution of malaria parasitaemia risk ranges from 36 to 71 % across the country while the median predicted prevalence is 59 %. The predicted parasitaemia map shows the higher risks in the Southwest, South-Central and the Eastern region of the country. The above mentioned regions coincide with the regions of country bearing the highest vegetation density and receiving the highest annual rainfall relative to the northern part which receives less rainfall and is more “desertic”. The predicted parasitaemia risk map shows similarities as well as discrepancies with the previous mapping efforts. Compared to the *P. falciparum* endemicity map of the Malaria Atlas Project (MAP), common patterns were found in the northern and northeastern parts of the country, which appeared to be less burdened [40]. Discrepancies were identified regarding the highest burden areas which MAP places in the northwestern part of the country while our map estimates in southwestern Burkina Faso. Samadoulougou et al. [6] indicated that the northern and southwestern regions have the highest and lowest malaria risk respectively. The above discrepancies may be explained by the different climatic/environmental and other covariates used in the predictive models.

This study estimated higher number of infected children in the cities despite the relatively low prevalence observed in the urban settings. This finding is consistent with the results from previous study that used the BFDHS-MICS 2010 data [6]. Differences were observed between raw and population-adjusted parasitaemia risk estimates which is explained by the low prevalence observed in densely populated areas. For example the province of Kadiogo, one of the smallest provinces with the highest population density and the lowest population-adjusted raw parasitaemia risk (34 %), shows an even lower parasitaemia risk adjustment for the population (4.04 %). Similar results were found using Senegal MIS 2008 data [4].

An increasing risk gradient with age was found. Infants had the lowest risk while older children had the highest risk. An association was observed between socio-economic status and malaria risk, with children within the least poor quintile being substantially at reduced risk. Similar results were observed in a previous study in Burkina Faso and in other malaria endemic areas [4, 6].

## Conclusions

This study provides estimates of the effects of malaria interventions at country as well as at local scale. The estimated risk and intervention effect maps are valuable tools for identifying high-risk areas and areas with less effective interventions in order to improve malaria control in Burkina Faso. These outputs can serve as benchmarks to evaluate the effectiveness of future control interventions and progress of the efforts towards disease control.

## Additional files

10.1186/s12936-016-1282-x Title: Modelling details. Description: The file provided represents details about models formulation.
10.1186/s12936-016-1282-x Title: Map of nighttime land surface temperatures (LSTN). Description: The file provided represents the pattern of nighttime land surface temperatures.
10.1186/s12936-016-1282-x Title: Map of the distance between the clusters and the nearest rice-paddy field in kilometer. Description: The file provided represents the distance between the clusters and the nearest rice-paddy field.

## Abbreviations

ITN: insecticide-treated bed nets
IRS: indoor residual spray
ACT: artemisinin-based combination therapy
BFDHS-MICS 2010: Burkina Faso Demographic and Health Survey-Multiple Indicator Cluster Survey
MIS: Malaria Indicator Survey
GMAP: global malaria action plan
IPTp: intermittent preventive treatment for pregnant women
INSD: National Institute for Statistics and Demography
RDT: rapid diagnostic test
ITNpU5: the number of ITNs per child under 5 years
ITNsU5: the proportion of children under five who slept under an ITN the night preceding the survey
ITNpPR: the total number of ITNs per household member
SES: socioeconomic status
RS: remotely sensed
LSTD: day land surface temperature
LSTN: night land surface temperature
RFE: rainfall estimates
NDVI: normalized difference vegetation index
BCI: Bayesian credible interval
