# Supplementary material for: Assessing the effects of malaria interventions on the geographical distribution of parasitaemia risk in Burkina Faso
Source: Malar J. 2016 Apr 21;15:228. doi: 10.1186/s12936-016-1282-x (PMC4839146; doi:10.1186/s12936-016-1282-x)
Supplement: Supplementary file 1 — 10.1186/s12936-016-1282-x Title: Modelling details. Description: The file provided represents details about models formulation. [file 12936_2016_1282_MOESM1_ESM.docx]

**Appendix**

Let indicate the malaria parasitaemia status of child at location. We assume that follows a Bernoulli distribution and is related to its predictors using a logistic regression model, that is, , where is the risk of child at location of having malaria parasites, $\beta=(\beta_{0},\beta_{1},...,\beta_{K})^{T}$ is the vector of the$K$regression coefficients*.* Spatial correlation is taken into account by adding location-specific random effects modelled by a Gaussian process, with variance-covariance matrix related to an exponential correlation function between locations, that is , where is the Euclidian distance between location and, is the geographic variability and is a smoothing parameter that controls the rate of correlation decay with increasing distance. Bayesian geostatistical models fitted via Markov Chain Monte Carlo (MCMC) simulation were employed for parameter estimation and predictions. The specification of the Bayesian hierarchical model requires prior distribution for all model parameters. The spatial correlation parameters and were assigned an inverse gamma and a gamma prior respectively, and.

Bayesian variable selection was carried out to identify the best set of predictors and their functional form using a variable selection approach known as stochastic search[38]. In particular, for each predictorwe introduce a categorical indicator parameter $I_{p}$suggesting exclusion of the predictor from the model ($I_{p}=0$), inclusion in linear ($I_{p}=1$) or categorical form ($I_{p}=2$). $I_{p}$has a probability mass function $\prod_{j=0}^{2} \pi_{j}^{\delta_{j}(I_{p})}$ where $\pi_{j}$ are the inclusion probability of functional form *j* (i.e. ) such that $\sum_{j=0}^{2} \pi_{j}=1$ and $\delta_{j}(\cdot)$is the Dirac function, $\delta_{j}(I_{p})=\left\{\begin{matrix} 1 & if\text{ }I_{p}=j \\ 0 & if\text{ }I_{p}\neq j \end{matrix}\right.$. Furthermore, we assume a spike and slab prior for the corresponding regression coefficient. For the coefficient $\beta_{p}$of the predictor in linear form we take $\beta_{p}\sim\delta_{1}(I_{p})N(0,\tau_{p}^{2})+\left(1-\delta_{1}(I_{p})\right)N(0,\upsilon_{0}\tau_{p}^{2})$ proposing a non-informative prior for $\beta_{p}$in case is included in the model in linear form (slab) and an informative normal prior shrinking $\beta_{p}$ to zero (spike) if is excluded from the model. Similarly, for the coefficient $\left\{\beta_{p,l}\right\}_{l=1,...L}$ corresponding to the categorical form of with $L$categories, we assume that$\beta_{p,l}\sim\delta_{2}(I_{p})N(0,\tau_{p,l}^{2})+\left(1-\delta_{2}(I_{p})\right)N(0,\upsilon_{0}\tau_{p,l}^{2})$. For the inclusion probabilities, we adopt a non-informative Dirichlet distribution with hyper-parameter $\alpha=(1,1,1)^{T}$that is,

$\pi=(\pi_{0},\pi_{1},\pi_{2})^{T}\sim Dirichlet(3,\alpha)$.

Age and socioeconomic quintiles were not part of the variable selection. Continuous covariates were standardized in order to acquire better correlation properties and reduce the Markov chain Monte Carlo simulation (MCMC) computational time[39].

Model parameters were estimated using Markov chain Monte Carlo simulation (Gibbs sampling)[40]. Starting with some initial values about the parameters, two chains sampler were run discarding the first 5000 iterations. Convergence was assessed by Gelman and Rubin diagnostic[41] and kernel density plots.

**Estimating the effect of intervention at sub-national (health district) level**

The above model was extended to include intervention coverage measures with spatially varying coefficients as follows: , where $Z_{i}^{(m)}(A_{j})$is the $m$ intervention coverage measure aggregated over the health district $A_{j}$of the $s_{j}$location, $b_{mj}$is the corresponding spatially varying coefficient (i.e. effect of intervention at $A_{j}$district) and$M$ is the number of spatially varying interventions. Gaussian conditional autoregressive (CAR) prior distributions were assumed for the $b_{m}$, that is $b_{m}\sim N(b_{m0}1,\Sigma_{m})$where $b_{m0}$is the overall effect of the $m$intervention at country level and $\Sigma_{m}^{-1}=\sigma_{m}^{-2}(D-W)$, $D$ is a diagonal matrix with entries, the sum of the neighbours of each health district, $W$is a proximity matrix.
