# Supplementary figures and images for: Assessing the effects of malaria interventions on the geographical distribution of parasitaemia risk in Burkina Faso
Source: Malar J. 2016 Apr 21;15:228. doi: 10.1186/s12936-016-1282-x (PMC4839146; doi:10.1186/s12936-016-1282-x)

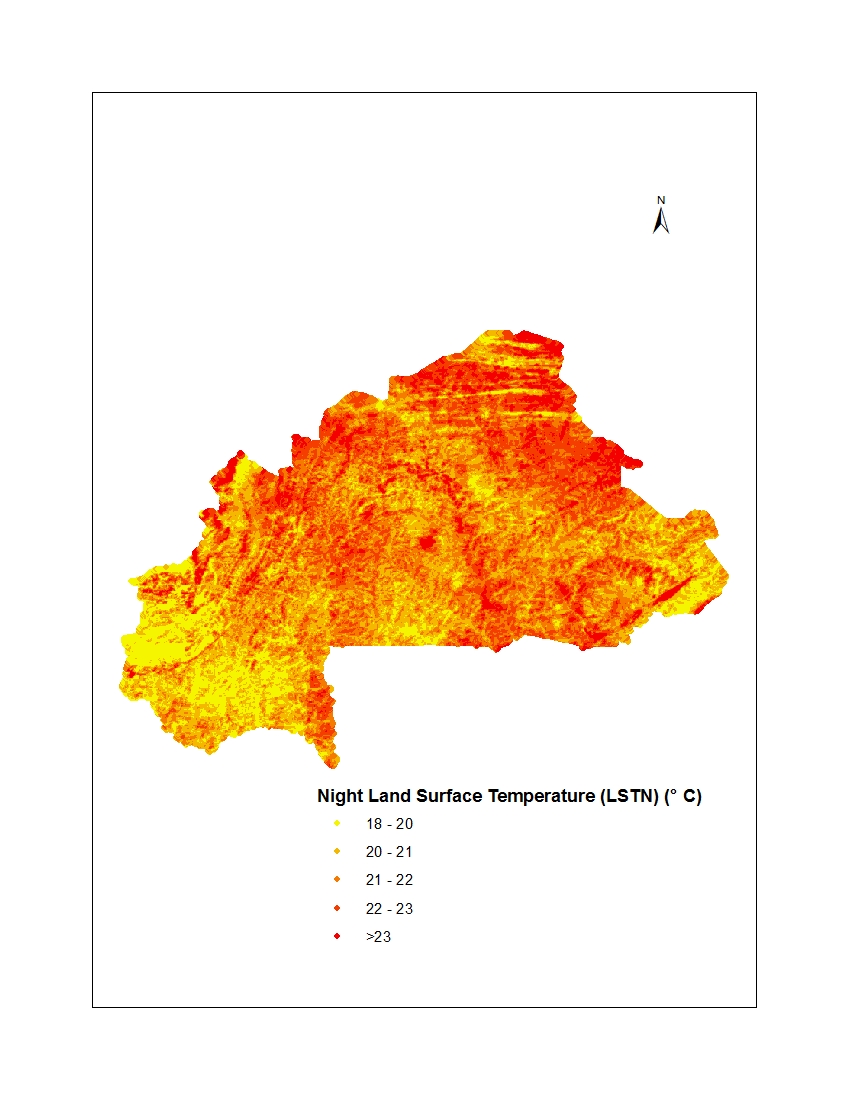


Figure 1: Night Land Surface Temperature (LSTN)

Supplement: Supplementary file 2 — 10.1186/s12936-016-1282-x Title: Map of nighttime land surface temperatures (LSTN). Description: The file provided represents the pattern of nighttime land surface temperatures. [file 12936_2016_1282_MOESM2_ESM.docx]

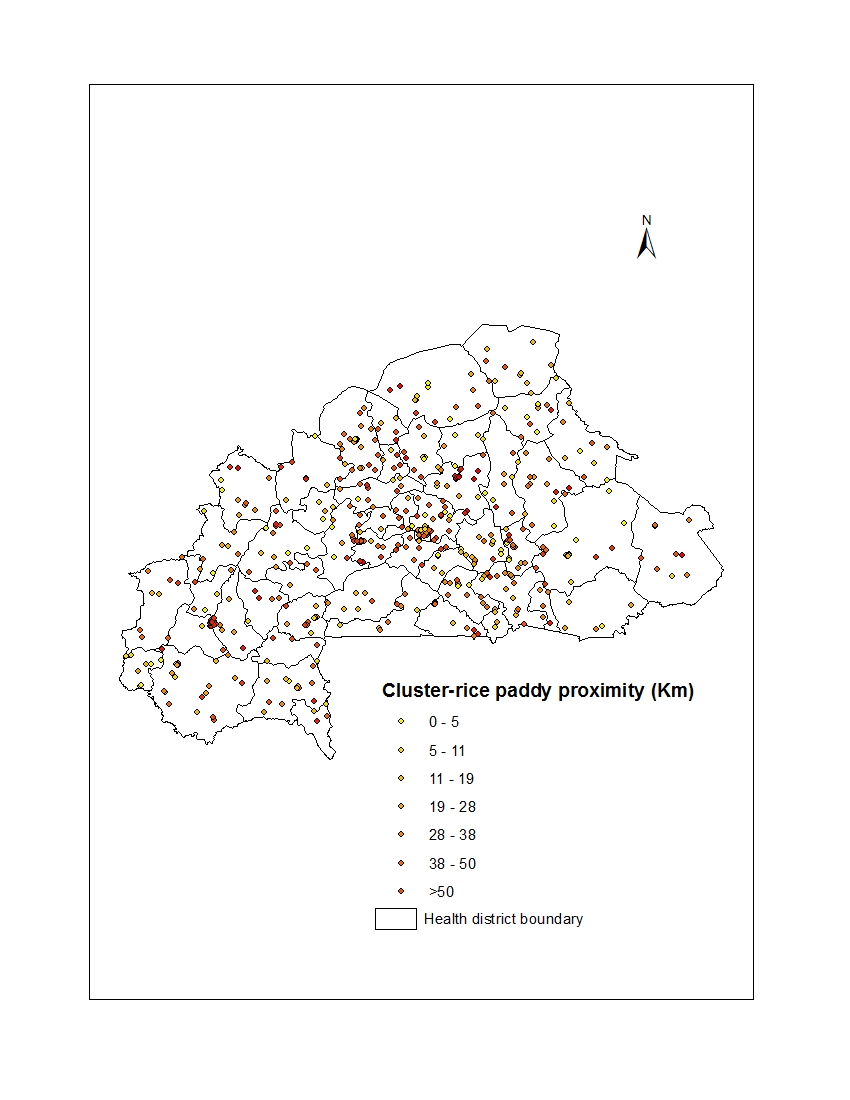


Figure 2: Map of the distance between the clusters and the nearest rice-paddy field in kilometer

Supplement: Supplementary file 3 — 10.1186/s12936-016-1282-x Title: Map of the distance between the clusters and the nearest rice-paddy field in kilometer. Description: The file provided represents the distance between the clusters and the nearest rice-paddy field. [file 12936_2016_1282_MOESM3_ESM.docx]
